# Mediating Effects of Perceived Social Support on the Relationship between Comfort and Hope in Hospitalized Patients with Acute Ischemic Stroke

**DOI:** 10.1155/2024/6774939

**Published:** 2024-07-05

**Authors:** Yueyue He, Rui Wang, Linqi Mo, Ling Feng

**Affiliations:** ^1^ Department of Neurology West China Hospital Sichuan University, Chengdu, China; ^2^ West China School of Nursing Sichuan University, Chengdu, China

## Abstract

**Background:**

The relationship among comfort, perceived social support, and hope should still be further explored. Clarifying the relationship between the aforementioned variables can enable clinical staff to implement tailored and effective intervention strategies for enhancing the management and quality of care of patients with ischemic stroke.

**Aim:**

This study aims to investigate the relationship between comfort, perceived social support, and hope in hospitalized patients with acute ischemic stroke and to explore the mediating effect of perceived social support on comfort and hope.

**Methods:**

A correlational cross-sectional study was performed using an online questionnaire. The study was conducted from January to August 2023 among 572 patients with acute ischemic stroke, and finally 534 valid questionnaires were included in the analysis. The general information questionnaire, Modified Barthel Index, Shortened General Comfort Questionnaire, Perceived Social Support Scale, and Herth Hope Index were utilized for investigation. Mediation analysis was performed by structural equation modelling. Indirect effects were evaluated through bootstrapping. Data analysis was performed using the statistical program packages, namely, *SPSS 29.0* and *AMOS 24.0*.

**Results:**

The comfort, perceived social support, and hope scores of patients with acute ischemic stroke were 94.1 (11.92), 72.74 (10.26), and 40.55 (4.99), respectively. The participants' hope was positively related to comfort (*r* = 0.531, *p* < 0.001) and perceived social support (*r* = 0.589, *p* < 0.001). Perceived social support exerts a partial mediating role between comfort and hope, and the mediating effect was 0.159 (95% CI [0.117, 0.210]), accounting for 25.0% of the total effect.

**Conclusion:**

We reported that comfort—directly and indirectly—exerts a positive impact on hope. Particularly, perceived social support enhances the impact of comfort on hope; perceived social support mediates the relationship between comfort and hope. Clinical staff should correctly understand the relationship among the three variables; they should effects targeted strategies to enhance patient comfort and social support, thereby increasing the hope level among ischemic stroke patients and bolstering confidence in disease management. *Implications for Nursing Management*. This study demonstrates that comfort and perceived social support serve as protective factors for hope among ischemic stroke patients. This observation provides evidence supporting the optimization of management for ischemic stroke patients from the perspectives of the cognitive adaptation theory and comfort theory. The findings of this study contribute to a more optimal understanding among clinical caregivers regarding the mechanisms underlying the relationship between comfort, social support, and hope, and it facilitates the adoption of effective intervention strategies for promoting the psychological management of ischemic stroke patients and enhancing patient care quality.

## 1. Introduction

Stroke is the second leading cause of death and the third leading cause of disability worldwide, and exhibits high morbidity, disability, and mortality [1]. There are more than 2 million new cases of stroke in China every year, and stroke is the first leading cause of death and disability in adults [2]. Acute ischemic stroke (AIS) accounts for 60% to 80% of all strokes [3]. Stroke often exhibits an acute onset and rapid progression [4]. Due to its severity, unpredictability, and uncontrollability, patients become prone to neurological dysfunction, hemiplegia, anxiety and depression, and other physical and mental symptoms [5, 6]. They exhibit increased negative emotions and negative attitudes toward the disease and a crucially reduced hope level [7]. Patients require comfort to alleviate the psychological distress occasioned by these symptoms, as well as hope to treat the illness [8]. Hope can enable patients to rebuild personal confidence and can make patients believe that a positive, realistic, and expected life goal can be achieved [9]. As an internal strength of psychology, it can enable patients to overcome difficulties, relieve pain, and enhance confidence in overcoming the disease. The recovery of self-care ability in patients with acute ischemic stroke is a lengthy process that requires patients to be full of hope and to accept their condition and exhibit active psychological recovery to obtain more optimal results [10]. However, patients hope is influenced by many aspects, such as occupation, education level, and psychological status [11, 12]. The crucial factor is the patient's feeling about the disease (i.e., comfort and perceived social support), which is the source and motivation of hope [7, 13].

Comfort is a pleasant experience, a desired state of satisfaction, and a feeling of positivity and strength in one's ability to cope with crisis and challenge [14]. Enhanced comfort after therapeutic interventions may increase hope and confidence and facilitate healing [15]. Long-term cognitive comfort indicates that life is not dynamic, which can easily lead to cognitive habits, thereby creating a scenario in which individuals fall into cognitive comfort zones and, thus, lose interest in life [16]. Patients with acute ischemic stroke often exhibit emotional, concentration, and fatigue problems, which generally transition from a state of cognitive stress to a state of cognitive relaxation and may fall into a cognitive comfort zone [17]. How to enable them to make decisions that can facilitate life- and cognitive environment-related changes is a novel experience and challenge for the mechanism of patients with acute ischemic stroke. Kolcaba defines comfort as “The immediate experience of being strengthened by having needs for relief, ease, and transcendence met in four contexts: physical, psychospiritual, sociocultural, and environmental, and it is so much more than the absence of pain” [18]. The patient relieved physical, psychological, and environmental discomfort; achieved spiritual comfort; and was treated as an individual with feelings, thoughts, values, and dignity [19]. Thus, comfort can promote patients with acute ischemic stroke to be able to express their emotions, identify the meaning and value of life, build confidence in the treatment of disease, and establish hope for survival and the future [20].

We examine perceived social support in the context of the transition to building hope, utilizing cognitive adaptation theory as a framework for conceptualizing resilience [21]. Social support enables individuals to feel loved, valued, and respected and includes family, friends, and other social supports [22]. It can also help individuals aspire to health, rebuild trust with others, and begin to integrate into social groups [23]. Optimal social support can provide individuals with trust to relieve stress, prevent anxiety, induce strength, and motivation in others to persevere and enhance personal hope and confidence in the future. In addition, studies have confirmed that there is a positive correlation between the comfort and hope felt by patients [24]. However, the relationship among comfort, perceived social support, and hope and their functional paths are unclear and should be further explored. From the perspective of the development of the positive psychology of hope, this study explores the influence path and structural causal relationship of comfort and perceived social support on the level of hope. Thus, it provides an entry point of psychological intervention for patients to actively participate in disease rehabilitation after increasing their hope and treatment confidence.

## 2. Methods

### 2.1. Study Design

This was a cross-sectional study and inferential analysis from West China Hospital, Sichuan University in Chengdu, Sichuan Province, China. This descriptive correlation study was designed to examine the relationship among comfort, perceived social support, and hope in hospitalized patients with acute ischemic stroke. A convenience sample was utilized to select patients from January to August 2023.

The sample size calculation method intended for this study was based on a total of 52 items across three scales, with the sample size being 10–15 times the total number of items [25, 26]. Considering potential incomplete data collection from patients, we increased the sample size by 10%, leading to a final calculated sample size of 572 cases. Questionnaires were distributed to a total of 572 patients who participated in this study, and surveys with invalid data and missing data were eliminated. Thirteen individuals who provided incomplete responses and 25 individuals who completed poor-quality questionnaires were excluded. Finally, a total of 534 valid questionnaires were included in the analysis, with an effective recovery rate of 93.4%.

### 2.2. Inclusion and Exclusion Criteria

The inclusion criteria were as follows: (1) age over 18 years; (2) AIS diagnosis made by neurologists and confirmed using computed tomography (CT) or magnetic resonance imaging (MRI); (3) consciousness and cognition were normal; and (4) patients could provide consent and were willing to cooperate with questionnaire completion.

The exclusion criteria were as follows: (1) incomplete clinical data; (2) previous history of other central nervous system diseases; (3) severe cardiac dysfunction, pulmonary dysfunction, liver dysfunction, or renal dysfunction; (4) acute embolism of the external cerebral artery; and (5) pregnancy.

### 2.3. Instruments

1. General information questionnaire. The general information questionnaire was utilized to collect demographic information: gender, age, level of education, marital status, provider payments, and comorbidity.
2. Shortened General Comfort Questionnaire (GCQ) [27]. This Likert 6 self-rating scale has 28 items; transcendence is addressed in four domains, namely, physical, psychospiritual, sociocultural, and environmental; and higher scores indicate better comfort. Examples of items relating to the four domains include, “I have a poor appetite” (physical), “My beliefs give me peace of mind” (psychospiritual), “My friends remember me with their cards and phone calls” (sociocultural), and “These surroundings are pleasant” (environmental). Negatively worded items are reverse scored. The GCQ exhibited a Cronbach's *α* of 0.88. The Chinese version exhibited reliability, with a Cronbach's *α* of 0.892 [28].
3. Perceived Social Support Scale (PSSS) [29]. The scale consists of support from family and friends and has 12 items; each item was scored on a 7-point Likert scale ranging from 1 (very strongly disagree) to 7 (very strongly agree), with a higher total score indicating a higher level of social support. The Chinese PSSS has been utilized among Chinese patients and has exhibited satisfactory reliability. Cronbach's *α* of the scale was calculated to be 0.962 [29].
4. Herth Hope Index (HHI) [9]. The scale is a 12-item summated rating scale designed as an individual measure of hope. Participants are asked to rate each item using a 4-point Likert response format. The internal consistency of the HHI in ill adults has been acceptable [30]. Cronbach's *α* coefficients for total HHI scores were 0.84 for stroke survivors [10].

### 2.4. Data Collection

The questionnaire was uploaded to the Questionnaire Star platform, an online crowdsourcing platform, and the survey was, subsequently, conducted through the WeChat app. The purpose and significance of the investigation were fully explained to the patients by the investigators. The survey was completed only after signing the informed consent form. Otherwise, they could not complete the questionnaire. The completed scales were immediately collected and initially reviewed by the investigator. If any omission was identified, the questionnaire was to be completed on the spot and checked again. Data were treated as invalid if subjects suddenly felt discomfort, leading them to not complete all questions. Incomplete and semifinished questionnaire data were eliminated and recorded and finally reviewed by two researchers for accuracy.

### 2.5. Ethical Considerations

The authors confirm adherence to ethical guidelines and obtained ethical approval (from the institutional review board). This study was performed in accordance with the ethical principles of the 1964 Declaration of Helsinki and it was approved by the Ethics Committee of West China Hospital, Sichuan University (No: 2022[1969]).

### 2.6. Statistical Analysis

Data analysis was conducted using the statistical program packages *SPSS 29.0* and *AMOS 24.0*. In the descriptive statistics, the mean, standard deviation, frequency, and percentage were reported. Pearson correlation was utilized to analyze the correlation between variables. *AMOS24.0* established a structural equation model (SEM) of the mediating role of perceived social support between comfort and hope. Comfort and perceived social support are the independent variables, and hope is the result variable. Moreover, subscale scores are utilized as the indicators for the latent factor. The maximum likelihood estimation method was utilized to estimate the model parameters, and the nonparametric percentile bootstrap method of bias correction was utilized to calculate the confidence interval of the effect. Bootstrapping was utilized to verify the mediation effect, with a duplicate sample size of 2000. If the 95% CI (confidence interval) of the indirect effect did not include zero, the mediation effect was proved to be significant [31]. We adopted many common indexes to evaluate the model in the current study, including the chi-square ratio to degrees of freedom (*x*^2^/ d*f*), root mean square error of approximation (*RMSEA*), goodness-of-fit index (*GFI*), normed fit index (*NFI*), incremental fit index (*IFI*), Tucker–Lewis index (*TLI*), and comparative fit index (*CFI*). If a model is acceptable, the *x*^2^/ d*f* should be less than 5, and other indexes should be greater than 0.90 [32]. A *p* value <0.05 was considered to be statistically significant.

## 3. Results

The mean age of the sample was 62.15 ± 15.03 years, including 369 males. The general characteristics of the patients are depicted in Table 1.

Correlation analysis was performed to verify the relationship between comfort, perceived social support, and hope. Consequently, as illustrated in Table 2, there were bivariate correlations between the subfactors of the main variables. The participants' hope was positively related to comfort (*r* = 0.531, *p* < 0.001) and perceived social support (*r* = 0.589, *p* < 0.001). The participants' perceived social support was also positively related to comfort (*r* = 0.383, *p* < 0.001). All relevant subcategories were positively associated with one another. In addition, most of the coefficients were below 0.80 (i.e., the standard for multicollinearity), indicating that there is no apparent multicollinearity problem [33].

**Figure 1:**
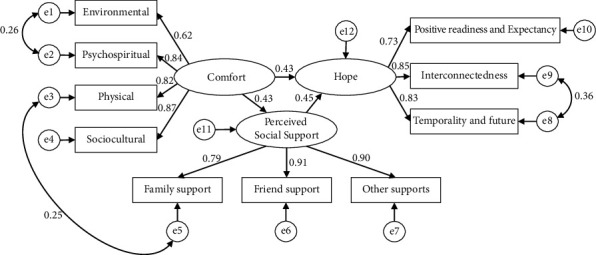
Path diagram for the hypothetical model.

To further explore the effects of comfort and perceived social support on Hope in patients with acute ischemic stroke, we tested for the mediating effects of perceived social support on comfort and hope. According to the research conducted by Wen and Fan [34], we tested this mediating model and constructed the structural equation model (SEM) using AMOS software. We modified the model by adding residual correlation according to the significant results of the initial model parameters and the model correction index provided by AMOS [35]. Herein, 3 model revisions were performed. The results of the SEM analysis indicated that this model was acceptable, *x*^2^/ d*f* = 1.722, *RMSEA* = 0.037 (95% *CI*: 0.020, 0.052), *GFI* = 0.978, *NFI* = 0.984, *IFI* = 0.993, *TLI* = 0.991, *CFI* = 0.993. The final structural equation model was depicted in Figure 1.

The mediation effect was tested using the bootstrap method with bootstrap samples of 2,000 (Table 3). The results indicated that the bootstrap 95% confidence intervals of the direct and indirect effects of comfort on hope did not contain 0. This observation indicated that perceived social support exerts a partial mediating role between comfort and Hope, and the mediating effect was 0.159 (95% CI [0.117, 0.210]), accounting for 25.0% of the total effect.

## 4. Discussion

Due to the characteristics of a high recurrence rate and high disability rate of stroke, most patients worry or even fear the future prognosis of the disease when treating it [36]. This study revealed that the score of hope in patients with acute ischemic stroke was 40.55 (4.99), which was slightly higher than that of 37.7 (4.46) in previous studies [10], which may be rationalized as follows: with the continuous progress of medical technology in recent years, an increasing number of treatment options have emerged, and the treatment effect of acute ischemic stroke may now be considerably enhanced compared with previous eras. This study mainly included hospitalized patients who were conscious, stable, and able to cooperate with the questionnaire, and most of these patients exhibited more optimal treatment outcomes than those with severe diseases. This observation indicates that we should consider the physical symptoms and signs in the acute stage, create a favorable communication environment and health education, and offer patients care and emotional support. Medical practitioners should, in turn, eliminate their negative emotions, help patients with a more positive and healthy mentality cope with the troubles occasioned by the disease, and promote the enhancement of the hope level in stroke patients.

The results of this study indicate that the comfort of patients with acute ischemic stroke is positively correlated with hope. Herein, the subjects are ischemic stroke patients, who are primarily elderly males. They generally exhibit a lower overall level of education, and more than half of these patients have one or more accompanying chronic illnesses. These patients face physiological, psychological, and environmental challenges amid the symptoms of the disease and emotional distress [37, 38]. The results of this study indicate that comfort serves as both a preceding variable and a critical intervention variable, aligning broadly with previous research outcomes [39, 40]. Changes in comfort levels impact patients' confidence in disease treatment and their expectations for future life [41]. More comfort and encouragement from healthcare professionals can assist patients in alleviating emotions and in prompting them to step out of their comfort zones [41]; thus, patients can face the challenges posed by the disease with a more positive attitude, which is beneficial for altering cognitive states, aiding in physical and mental recovery, and ultimately increasing hope for rehabilitation. This indicates that the effect of symptom relief is more prominent by evaluating the comprehensive state of patients and, subsequently, formulating and implementing various discomfort relief measures according to the needs and state of patients [42]. The relief of symptoms contributes to the relief of negative emotions and the improvement of the quality of life [43]. In this process, the patient's recovery confidence also increases, which can be reflected in the improvement of the hope level.

Perceived social support reflects the individual's emotional experience and satisfaction, accompanied by a feeling of being respected and supported in society, which is closely related to the individual's subjective feelings [44]. The results of this study revealed that perceived social support can significantly positively predict the level of hope, thereby indicating that higher levels of perceived social support are associated with higher levels of hope. Patients with higher perceived social support were more likely to establish favorable relationships with others, such as family and social relationships [45]. When individuals perceive more peer support, they promote the development of peer relationships, which makes individuals more likely to stimulate internal motivation and seek effective methods of addressing challenges when handling setbacks and pressure situations as a means of improving their hope level [46]. The surrounding environment of patients with acute ischemic stroke changes when they are admitted to the hospital with a sudden illness. They are more sensitive to interpersonal relationships around them and are more likely to exhibit favorable social relationships and reduce the occurrence of bad emotions such as depression when they receive objective supportive behaviors or support resources from others [47, 48]. When patients feel a more optimal interpersonal relationship, they may enhance their expectations for the future, set goals, and believe that they can achieve them, thereby improving their hope level.

The results of this study revealed that perceived social support exerts a partial mediating role in the effect of comfort on hope. The comfort of patients with acute ischemic stroke exerts a direct effect on hope, and it, simultaneously, exerts an indirect effect on hope through perceived social support. According to the buffering effect model of social support, favorable social support can enable patients with high stress to avoid or suffer fewer adverse effects occasioned by stressful events [49]. When patients with acute ischemic stroke are faced with disability occasioned by their disease, various dysfunctions can lead to great stress [50]. These physical and psychological pains all appear suddenly with the disease [51]. However, when patients are in a more comfortable atmosphere, they feel more social support from family, friends, and other aspects. Thus, the adverse impact of the disease on psychology can be weakened, and the patients can begin to actively cope with life and face the disease and have more confidence and hope for the treatment of the disease. This observation indicates that medical staff should not only consider the physical and psychological comfort of patients with acute conditions but also consider the impact of perceived social support on patients. The hope level of patients should be simultaneously enhanced from two aspects. Based on the path analysis of the mediating effect, the corresponding intervention plan was formulated to create a comfortable treatment process for patients and provide social support in many aspects. We can encourage patients to actively seek the help of family or friends to relieve economic or emotional pressure, reduce negative emotions, and finally face the disease and life with hope.

## 5. Conclusions

We reported that comfort, directly and indirectly, has a positive impact on hope. Particularly, perceived social support enhances the impact of comfort on hope; perceived social support mediates the relationship between comfort and hope. Clinical staff should correctly understand the relationship among the three variables, taking targeted strategies to enhance patient comfort and social support, thereby increasing the hope level among ischemic stroke patients and bolstering confidence in disease management.

## 6. Limitations

Due to the limited region and energy, this study adopted a cross-sectional survey, and the research object was obtained from a tertiary hospital; therefore, because the sample representativeness was limited, readers should be cautious when applying the findings to patients from other countries or different cultural backgrounds. Further studies should be conducted to expand the sample size and study population; thus, the conclusions of this study can be verified. Personalized interventions can also be formulated from the aspects of comfort and social support to increase the hope of patients.

## Figures and Tables

**Table 1:** General characteristics (*N* = 534).

Variable	Category	*n* (%)
Gender	Male	369 (69.1)
	Female	165 (30.9)

Level of education	Primary school or below	163 (30.5)
	Junior	128 (24.0)
	Senior	101 (18.9)
	College degree or above	142 (26.6)

Marital status	Unmarried	18 (3.4)
	Married	469 (87.8)
	Widowed or divorced	47 (8.8)

Provider payments	Medical insurance	493 (92.3)
	Self-paying medical	41 (7.7)

Comorbidity	Hypertension	335 (62.7)
	Diabetes mellitus	179 (33.6)
	Hyperlipidemia	64 (12.0)
	Heart disease	146 (27.3)
	Atherosclerosis	156 (29.2)

**Table 2:** Correlations between comfort, perceived social support, and hope (*N* = 534).

Variables	Mean (SD)	1	1-1	1-2	1-3	1-4	2	2-1	2-2	2-3	3	3-1	3-2	3-3
1. Comfort	94.1 (11.92)	1												
1-1. Physical	27.41 (3.82)	0.869^*∗∗*^	1											
1-2. Psychospiritual	31.59 (4.83)	0.923^*∗∗*^	0.695^*∗∗*^	1										
1-3. Environmental	11.49 (1.58)	0.711^*∗∗*^	0.493^*∗∗*^	0.631^*∗∗*^	1									
1-4. Sociocultural	23.61 (3.39)	0.888^*∗∗*^	0.705^*∗∗*^	0.741^*∗∗*^	0.576^*∗∗*^	1								

2. Perceived social support	72.74 (10.26)	0.383^*∗∗*^	0.405^*∗∗*^	0.308^*∗∗*^	0.168^*∗∗*^	0.371^*∗∗*^	1							
2-1. Family support	25.04 (3.29)	0.360^*∗∗*^	0.421^*∗∗*^	0.275^*∗∗*^	0.155^*∗∗*^	0.326^*∗∗*^	0.868^*∗∗*^	1						
2-2. Friend support	23.75 (4.09)	0.355^*∗∗*^	0.361^*∗∗*^	0.293^*∗∗*^	0.168^*∗∗*^	0.345^*∗∗*^	0.936^*∗∗*^	0.707^*∗∗*^	1					
2-3. Other supports	23.95 (3.83)	0.337^*∗∗*^	0.338^*∗∗*^	0.275^*∗∗*^	0.138^*∗∗*^	0.346^*∗∗*^	0.933^*∗∗*^	0.712^*∗∗*^	0.831^*∗∗*^	1				

3. Hope	40.55 (4.99)	0.531^*∗∗*^	0.556^*∗∗*^	0.442^*∗∗*^	0.286^*∗∗*^	0.474^*∗∗*^	0.589^*∗∗*^	0.632^*∗∗*^	0.518^*∗∗*^	0.480^*∗∗*^	1			
3-1. Temporality and future	13.40 (1.88)	0.522^*∗∗*^	0.520^*∗∗*^	0.445^*∗∗*^	0.309^*∗∗*^	0.471^*∗∗*^	0.514^*∗∗*^	0.558^*∗∗*^	0.452^*∗∗*^	0.415^*∗∗*^	0.907^*∗∗*^	1		
3-2. Positive readiness and expectancy	13.71 (1.80)	0.324^*∗∗*^	0.391^*∗∗*^	0.254^*∗∗*^	0.116^*∗∗*^	0.283^*∗∗*^	0.566^*∗∗*^	0.579^*∗∗*^	0.511^*∗∗*^	0.471^*∗∗*^	0.827^*∗∗*^	0.591^*∗∗*^	1	
3-3. Interconnectedness	13.44 (1.96)	0.555^*∗∗*^	0.560^*∗∗*^	0.469^*∗∗*^	0.328^*∗∗*^	0.498^*∗∗*^	0.489^*∗∗*^	0.546^*∗∗*^	0.419^*∗∗*^	0.392^*∗∗*^	0.922^*∗∗*^	0.813^*∗∗*^	0.625^*∗∗*^	1

^*∗∗*^ *p* < 0.001.

**Table 3:** Results for the total, indirect, and direct effects of perceived social support on comfort with hope as a mediator (*N* = 534).

Model pathways	Estimate	*SE*	95% CI		Effect proportion (%)
			Lower	Upper	
Total effect	0.636	0.031	0.569	0.692	
Indirect effect	0.159	0.024	0.117	0.210	25.0
Direct effect	0.477	0.037	0.402	0.544	75.0

SE = standard error, CI = confidence interval.

## Data Availability

The processed data are available from the corresponding author upon reasonable request.

## Abbreviations

AIS: Acute ischemic stroke
SEM: Structural equation model
*x* ^2^: Chi-square value
d*f*: Degrees of freedom
*x* ^2^/d*f*: Ratio of the chi-square to degrees of freedom
RMSEA: Root mean square error of approximation
GFI: Goodness-of-fit index
NFI: Normed fit index
IFI: Incremental fit index
TLI: Tucker–Lewis index
CFI: Comparative fit index.
